# Direct shape determination of intermediates in evolving macromolecular solutions from small-angle scattering data

**DOI:** 10.1107/S2052252518005900

**Published:** 2018-05-30

**Authors:** Petr V. Konarev, Dmitri I. Svergun

**Affiliations:** a Laboratory of Reflectometry and Small-angle Scattering, A. V. Shubnikov Institute of Crystallography of Federal Scientific Research Centre ‘Crystallography and Photonics’ of Russian Academy of Sciences, Leninsky pr. 59, Moscow 119333, Russian Federation; b National Research Centre ‘Kurchatov Institute’, Akademika Kurchatova pl. 1, Moscow 123182, Russian Federation; c Hamburg Outstation, European Molecular Biology Laboratory, Notkestrasse 85, Hamburg 22607, Germany

**Keywords:** SAXS, *DAMMIX*, intermediates, macromolecular solutions, biological processes

## Abstract

Many important biological processes like amyloid formation, viral assembly *etc.* can be monitored *in vitro*. Small-angle X-ray scattering (SAXS) is one of the most effective techniques to structurally characterize these processes in solution. For monodisperse systems and some oligomeric mixtures, low-resolution shapes can be determined *ab initio* from the SAXS data, but for evolving systems, such analysis is hampered by the presence of multiple species and no direct reconstruction procedures are available. The authors consider a frequently occurring case where the scattering from the initial and final states of the process are known but there exists a major (unknown) intermediate component. A method is presented to directly reconstruct the low-resolution shape of this transient component together with its volume fractions from multiple scattering patterns recorded from an evolving system. The method is implemented in the computer program *DAMMIX* freely available to academic users and its effectiveness is illustrated in several synthetic and experimental examples.

## Abstract 2

The low-resolution shape of an unknown intermediate in an evolving nanostructured system is directly reconstructed from a global fit of the solution scattering data recorded during the process.

## 1. Introduction   

One of the most important and challenging problems in modern structural biology is the characterization of complex and evolving systems depicting dynamic biological processes. These systems can be analysed *in vitro* but high-resolution methods like X-ray crystallography, nuclear magnetic resonance or electron microscopy have limitations in the studies of such complicated and heterogeneous objects that change with time. Small-angle X-ray scattering (SAXS) (Svergun *et al.*, 2013 ▸) provides structural information on macromolecular systems in close to physiological solutions with high temporal resolution. For monodisperse solutions containing single purified species, methods to interpret the data in terms of three-dimensional models are well established and widely used. These include both low-resolution *ab initio* shape reconstruction and hybrid modelling techniques utilizing domains or subunits with known or predicted high-resolution structures to construct composite models [see, for example, a review by Mertens & Svergun (2010 ▸)]. Evolving systems, however, typically exist as equilibria of multiple species or states and the measured intensity contains contributions from all these species. A typical task for such multicomponent systems lies not in the reconstruction of the structure but in the determination of the volume fractions of the components. The scattering from the individual components is either known *a priori* or may be parameterized with simple shaped components (Konarev *et al.*, 2003 ▸). Examples of evolving systems studied by SAXS are given by amyloid fibril formation (Vestergaard *et al.*, 2007 ▸; Giehm *et al.*, 2011 ▸), maturation of virus-like particles (Matsui *et al.*, 2010 ▸; Aramayo *et al.*, 2005 ▸) and dynamic oligomeric equilibria (Xu *et al.*, 2008 ▸; Chiara *et al.*, 2013 ▸).

Several approaches are available to extract the scattering curves and abundances of components from the data on evolving systems, each of these approaches has advantages and limitations. For monomer–dimer mixtures, an MCR–ALS (multivariate curve resolution–alternating least-squares) analysis (Blobel *et al.*, 2009 ▸) was proposed, whereas for monomer–multimer mixtures the shape of the oligomeric species could be reconstructed by dummy residues (Petoukhov *et al.*, 2012 ▸). However, the former approach is applicable only to simple monomer–dimer equilibria, whereas the latter (implemented in the program *GASBORMX*) is restricted to dissociating proteins and could not be employed for nucleic acids or non-biological species (*e.g.* nanoparticles). Recently, a chemometric decomposition method was proposed and applied to amyloid fibril formation data (Herranz-Trillo *et al.*, 2017 ▸) utilizing an MCR–ALS procedure on differently weighted SAXS data sets. This method, implemented in the program *COSMICS*, does not require knowledge about the scattering contributions from the pure species, and, under some constraints based on the physical nature of the system, estimates of the scattering curves from the components and their relative abundances can be obtained. An evolving factor analysis (EFA) (Hopkins *et al.*, 2017 ▸) is another important recent development allowing one to separate overlapping peaks from elution profiles of online size-exclusion chromatography (SEC-SAXS) data and restore the scattering patterns from the separated components.

Several approaches were also proposed for selecting subsets from ensembles of possible conformers. These include, for example, an ensemble optimization method (EOM) (Bernadó *et al.*, 2007 ▸; Tria *et al.*, 2015 ▸) to select the best subset of models from a large ensemble pool or a constrained maximum-likelihood approach for the estimation of relative abundancies (Onuk *et al.*, 2015 ▸). These methods, however, require that the (tentative) models are pre-generated in advance.

Very often in studies of evolving systems, the initial and final states of a process (at its beginning and at the end) are stable and the SAXS patterns from these states can be reliably measured (or well approximated by theoretical scattering). During the process, a major intermediate is formed, and this intermediate structure disappears at the end such that its scattering, and therefore shape, cannot be directly retrieved from the experimental data. Instead, multiple scattering curves from the system are collected representing varying mixtures of different states. The presence of the intermediate can be detected with model-independent approaches like singular value decomposition (SVD), which indicate that more than two independent components (in the case of a clearly defined intermediate, three components) are required to describe the collected data (Golub & Reinsch, 1970 ▸). However, SVD does not allow one to extract the scattering pattern of the intermediate and therefore make conclusions about its structure. Here, we present a method to directly restore the shape of the unknown intermediate in an evolving system together with the volume fractions of the components at all recorded states. The method is based on the joint analysis of multiple data sets and it yields the best overall fit to all available scattering data. Its performance is illustrated on several synthetic and experimental data sets.

## 2. Shape reconstruction of an unknown component of an evolving system   

Let us have *K* scattering curves collected from an evolving system (*e.g.* kinetic time-resolved measurements). In the beginning, the system yields the intensity [*I*_m_(*s*), *e.g.* monomeric species]; at the end, there is a final defined state [*e.g.* large aggregate *I*_a_(*s*)]. The two scattering intensities *I*_m_(*s*) and *I*_a_(*s*) are assumed to be known, but during the time course of the reaction, an intermediate component is formed whose structure and scattering curve *I*_i_(*s*) are unknown. The scattering intensity at the *k*th point of the reaction is a linear combination
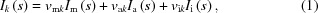
where *v*_m*k*_, *v*_a*k*_, and *v*_i*k*_ are the volume fractions of the components, *v*_m*k*_ + *v*_a*k*_ + *v*_i*k*_ = 1.

If the scattering pattern from the intermediate component *I*_i_(*s*) could be directly measured, its low-resolution shape could be reconstructed *ab initio*, *e.g.* with dummy-atom modelling (DAM). Here, the search space is represented by a densely packed grid of small spheres (dummy atoms) of sufficiently small radius *r* << *R*, where *R* is the characteristic particle size. Each sphere can belong either to the particle (index = 1) or to the solvent (index = 0). The scattering intensity from the DAM configuration *I*_DAM_(*s*) is rapidly calculated using spherical harmonics (Stuhrmann, 1970 ▸). A simulated annealing (SA) algorithm (Kirkpatrick *et al.*, 1983 ▸) is used for finding the optimal shape (*i.e.* the vector *X* containing the phase descriptions) by randomly changing one dummy atom per move to ultimately minimize the energy function
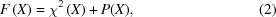
where *χ* ^2^(*X*) is the discrepancy between the experimental and calculated curves and *P*(*X*) is a penalty ensuring that the DAM is physically sensible (compact, interconnected, with the centre of mass close to the origin and, if applicable, also having proper anisometry). The method is implemented in the programs *DAMMIN *(Svergun, 1999 ▸) using the shape search in a limited space with the maximum diameter 2*R* and *DAMMIF* (Franke & Svergun, 2009 ▸) using unrestricted search space. The programs are routinely employed by thousands of researchers for *ab initio* shape reconstruction from SAS data.

For an evolving system, *I*_i_(*s*) cannot be measured; instead, the information about the intermediate component intensity is encrypted in the measured data through equation (1). We propose here a generalized method to utilize this information and reconstruct the shape of the intermediate together with the unknown volume fractions of the three components by simultaneously fitting all the experimental scattering patterns *I*_*k*_(*s*), *k = 1…K*.

The generalized function to be minimized *F*(*X*) is calculated as
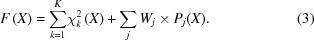
Here, the first term is the overall discrepancy between the experimental and calculated data over *K* experimental curves. The second term contains the physical constraints similar to those for *DAMMIN*/*DAMMIF* in equation (2) and includes the looseness penalty that demands the compactness of the model as well as the geometrical centre and radius of gyration (*R*_g_) penalty keeping the model close to the origin (Franke & Svergun, 2009 ▸). Additionally, this term contains a minimum fraction penalty that ensures that the average volume fraction of the intermediate (unknown) component should not become less than 5% to avoid meaningless solutions with marginal contribution to the measured data. The penalty weights (*W_j_*) balance between the discrepancy and physical constraints of the models. The default weights for the looseness and centre/*R*_g_ terms are the same as in *DAMMIF*, and the default value for the volume fraction penalty is equal to 0.5. The default values, similar to *DAMMIF*, work well in all tested cases, but they can be manually changed by the user if needed.

The shape is reconstructed using the algorithmic principle of *DAMMIF*, without limitations on the search space. The method suggests the bead size ensuring adequate representation of relatively small intermediates, like oligomers of the initial state. As an initial approximation for the intermediate, a sphere with the volume [(*V*_m_ + *V*_a_)/2] is selected and SA is employed to find the shape minimizing *F*(*X*) in equation (3). Given that the search space is unlimited and to avoid obtaining too large shapes ‘competing’ with the scattering from the final state, the volume of the intermediate model is restricted (by default, to be no larger than 50 times the volume of the monomer). At each SA step, the shape is randomly modified like in *DAMMIF*, the intensity *I*_i_(*s*) is recalculated and the volume fractions determined, yielding the best overall fit to the measured data. For each data set this is performed by fitting the experimental data using equation (1) with non-negativity constraints on the volume fractions [similar to the program *OLIGOMER* (Konarev *et al.*, 2003 ▸)]. The above algorithm is implemented in the computer program *DAMMIX* and the results of its application are presented below.

## 3. Applications to simulated and practical cases   

### 3.1. Simulated examples   

**Figure 1:**
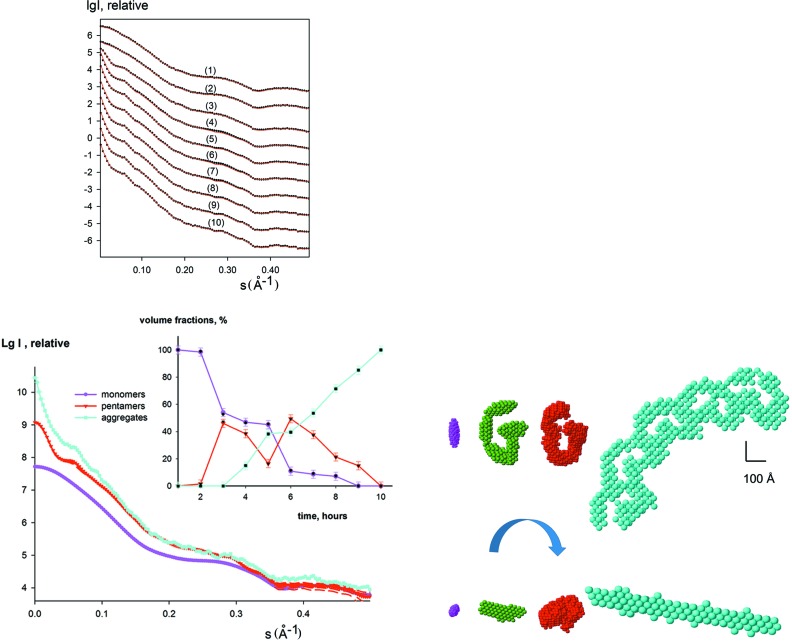
Simulated scattering curves from an evolving system (initial state, an ellipsoid; intermediate state, G-like structure from five ellipsoids; and final state, aggregate composed from five G-like intermediates). The pure species curves from the bead models were calculated using *DAMMIN*, the linear combinations with the ‘designed’ volume fractions were generated using *PRIMUS* (Konarev *et al.*, 2003 ▸). A relative error of 2% was added to the simulated data. In the top left panel, the simulated data are shown as dots, the fits as red solid lines. The shapes of the components (ellipsoid, G-structure, aggregate) are shown in the bottom right panel with magenta, green and cyan beads, respectively. A typical restored shape of the intermediate by *DAMMIX* is displayed in the bottom right panel with red beads. The scale bar is 100 Å. The scattering curves from the components are shown in the bottom left panel (the two most different restored curves for the intermediate obtained from multiple *DAMMIX* runs are shown with dashed red lines) and their restored volume fractions are displayed as an inset (the colours are the same as for the bottom right panel) together with the actual volume fractions used in the modelling (black; the error bars of volume fractions display the average dispersion over multiple *DAMMIX* runs).

The method was first tested on simulated data describing the processes of particle association. In the example presented in Fig. 1 ▸, we generated a system of ellipsoid-like particles and calculated ten synthetic curves as linear combinations from the scattering by an ellipsoid with semi-axes 20, 30 and 70 Å (corresponds to the initial ‘monomeric’ state), a G-like structure composed from five ellipsoids (corresponds to the intermediate ‘oligomeric’ state) and an aggregate composed from five G-like structures (the final ‘aggregate’ state of the system). Using the simulated data containing 2% relative error and the computed scattering curves from the monomers and aggregates, *DAMMIX* restored the G-like appearance of the intermediate state and obtained the volume fractions of the components within 3% compared with the ideal values (Fig. 1 ▸, inset). The model variability was assessed by the normalized spatial discrepancy (NSD) using the program *SUPCOMB* (Kozin & Svergun, 2001 ▸) in a similar way to *DAMMIN*/*DAMMIF* restorations (Franke *et al.*, 2017 ▸). An average NSD value was computed over 15 individual *DAMMIX* runs utilizing different random generations to be 〈NSD〉 = (0.92 ± 0.08); given that NSD about unity corresponds to a good correlation between shapes, this result points to a reproducible reconstruction. The comparison procedure also automatically selects the most typical reconstruction (the shape that has the best overlap, *i.e.* the minimum average NSD against all other models), and the most typical reconstructions are presented in Fig. 1 ▸ and subsequent figures. The variation of the restored volume fractions between individual runs stays within 3%, the oligomerization number of the intermediate component is close to the expected pentamer, and the deviations between the restored curves of the intermediate are within the errors (see Table 1 ▸). These results confirm the reproducibility and robustness of the shape reconstruction of the intermediate.

Several other tests on different simulated data were conducted and for all these synthetic examples *DAMMIX* restored the intermediates with high reliability. As an illustration, Fig. S1 (see supporting information) presents the results on a sphere–cylinder–prism system emulating lateral aggregation of intermediates, where *DAMMIX* successfully depicted the cylindrical shape of the intermediate and volume fractions of the components. The performance of the method in practical examples is especially interesting. In the following, applications of *DAMMIX* to the experimental data from evolving systems are presented and compared with the previously published results.

### 3.2. Insulin fibrillation   

**Figure 2:**
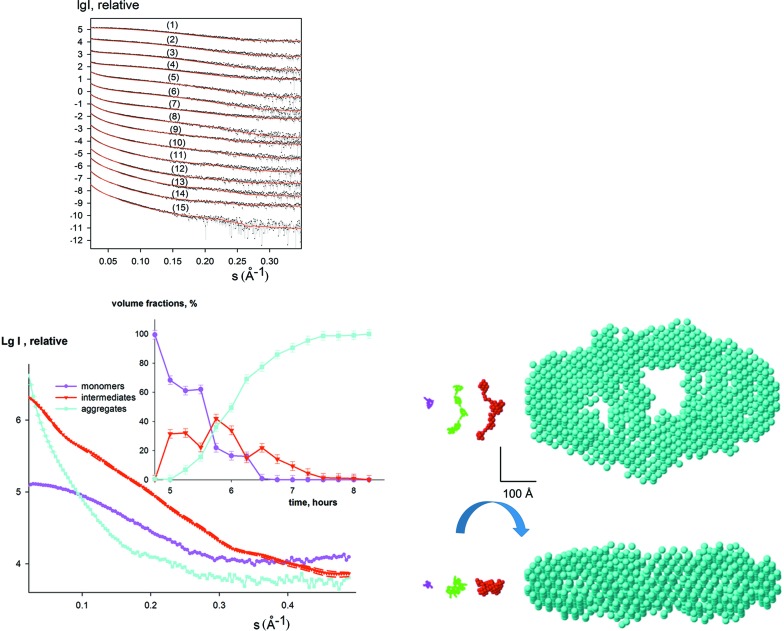
Analysis of the intermediate during insulin fibrillation. The initial state is well represented by a monomeric model of insulin (PDB entry 1guj) and the final state is approximated by the bead model of the mature fibril (Vestergaard *et al.*, 2007 ▸). The top left panel displays the experimental data (dots with error bars), and the *DAMMIX* fits as red solid lines. The shapes of the monomer and the aggregates are shown in the bottom right panel with magenta and cyan beads, respectively; the previously reported intermediate pentameric model is displayed with a green C_α_ trace (Vestergaard *et al.*, 2007 ▸). The restored shape of the unknown intermediate obtained by *DAMMIX *is displayed in the bottom right panel (red beads). The scale bar is 100 Å. The scattering curves from the components are shown in the bottom left panel (the two most different restored curves for the intermediate obtained from multiple *DAMMIX *runs are shown with dashed red lines) and their restored volume fractions are displayed as an inset; the colours are the same as the bottom right panel and the error bars of volume fractions display the average dispersion over multiple* DAMMIX *runs.

Amyloid fibrillation is a nucleation-dependent process and characterization of the nuclei is extremely important to understand its mechanism. The kinetic SAXS data collected during insulin fibrillation (Vestergaard *et al.*, 2007 ▸) include 15 experimental curves measured during the 4 h elongation phase that started after a 5 h incubation period (Fig. 2 ▸). The data cannot be represented by linear combinations of the monomers (initial state) and the mature fibrils (final state). The SVD analysis confirms that there are three components significantly contributing to the scattering signals during the fibril elongation.

The shape of the intermediate insulin component, the volume fractions of the monomers, intermediates and mature fibrils obtained by *DAMMIX* and the fits to the experimental data are displayed in Fig. 2 ▸. Interestingly, the reconstructed shape of the intermediate is an elongated structure showing several blobs on a string compatible with insulin monomers. This shape displays a remarkable agreement with the hypothetical model of insulin oligomer proposed by Vestergaard *et al.* (2007 ▸), which was constructed from monomeric insulins by an iterative modelling procedure. The *ab initio* reconstruction of the intermediate obtained without any *a priori* information corresponds very well to the earlier model and lends further support to the hypothesis that the oligomeric nucleus is the primary elongating unit of insulin amyloid fibrils. The stability of the results in repetitive *DAMMIX *runs is presented in Table 1 ▸.

### 3.3. Multiple assembly states of lumazine synthase   

The ability of proteins to form different quaternary structures is essential for many biological processes such as signal transduction, cell-cycle regulation and enzyme catalysis. An example is lumazine synthase that catalyses the penultimate step of riboflavin biosynthesis. Multiple assembly states were shown to be a general feature of this system by a joint SAXS and cryo-EM study (Zhang *et al.*, 2006 ▸). Lumazine synthase forms smaller capsids with a diameter of 160 Å (t1 capsids) and larger capsids with diameters of around 300 Å (t3 capsids). The relative abundance of small and large capsids is strongly dependent on buffer and pH as well as mutations. It was shown that, in addition to the two capsids, free facets from the dissociated or incomplete capsids may also be present in solution and their volume fractions were evaluated using the program *MIXTURE* (Zhang *et al.*, 2006 ▸). Therefore, the system can be described by three independent components.

**Figure 3:**
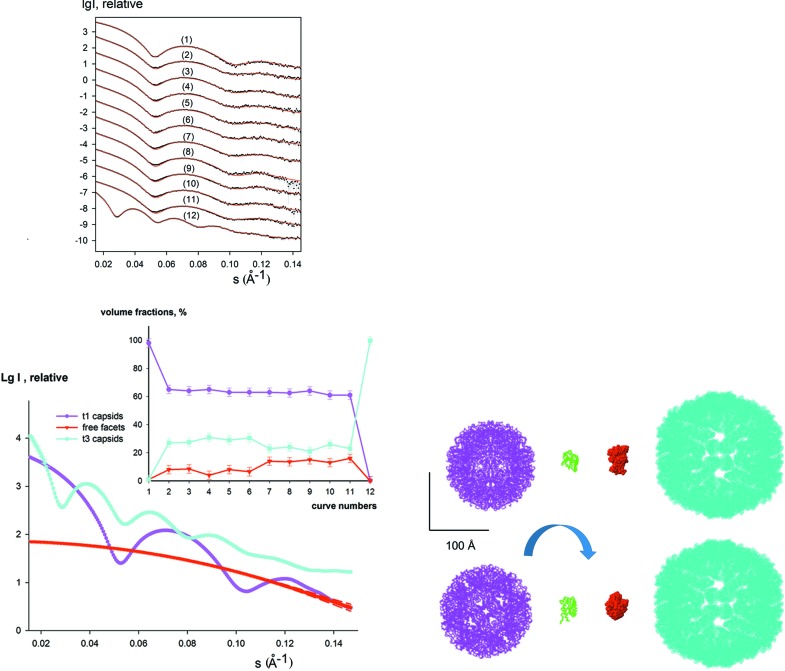
Intermediate detection in lumazine synthase capsid formation. The top left panel displays the data measured at different conditions [curve 1, wild type lumazine synthase from *B. subtilis* (LSBS) in borate buffer (pH 7.0); curves 2–6, wild type lumazine synthase from *A. aeolicus* (LSAQ) in phosphate buffer (pH 6.0, 6.5, 7.0, 7.5, 8.0); curves 7–11, LSAQ in Tris buffer (pH 7.0, 7.5, 8.0, 8.5, 9.0); curve 12, LSAQ mutant in Tris buffer (pH 8.0)]. The experimental data are shown as dots with error bars and the *DAMMIX* fits as red solid lines. Known ‘initial’ and ‘final’ states, t1 capsids of diameter 160 Å and t3 capsids of diameter of 300 Å, respectively, are shown in the bottom right panel with magenta and cyan beads. The experimental data from LSBS in borate buffer and LSAQ mutant in Tris buffer (curves 1 and 12, respectively) corresponding to these models were used as input in *DAMMIX* after regularization by *GNOM* (Svergun, 1992 ▸). The monomeric lumazine synthase (PDB entry 1rvv) is shown with green C_α_ traces, and a typical restored shape of the unknown component (dissociated fragments of capsids) obtained by *DAMMIX* is displayed with red beads. The scale bar is 100 Å. The scattering curves from the components are shown in the bottom left panel (the two most different restored curves for the intermediate obtained from multiple *DAMMIX* runs are shown with dashed red lines) and their restored volume fractions are displayed in the inset; the colours are the same as the bottom right panel and the error bars of volume fractions display the average dispersion over multiple *DAMMIX* runs.

The experimental data set analysed by *DAMMIX* contained a total of 12 scattering curves from the wild-type and mutant forms of lumazine synthase from *Aquifex aeolicus* data in phosphate (pH 6.0–8.0) or Tris (pH 7.0–9.0) and wild-type lumazine synthase from *Bacillus subtilis* in borate buffer (pH 7.0) with a maximum abundance of t1 capsids, and its mutant form in Tris buffer (pH 7.0) with predominantly t3 capsids (Fig. 3 ▸). *DAMMIX* allowed one to fit the entire data set and restore the shape of the dissociated fragments as well as the volume fractions of all the components in solution. The restored component displays the shape and size very similar to that of the lumazine synthase monomer (Fig. 3 ▸, right panel), and the volume fractions of the monomer agree well with the previously reported results (Fig. 3 ▸, inset). This example shows the potential of *DAMMIX* in retrieving the shapes of unknown components for systems forming multiple assembly states (*e.g.* virus-like particles or nanoparticles stabilized by polymer chains). The degree of model variability and average dispersion of the volume fractions over multiple *DAMMIX *runs are summarized in Table 1 ▸.

### 3.4. Concentration-dependent NGF oligomerization equilibrium   

With the proposed method it is also possible to study two-component equilibria (*e.g.* monomer–multimer mixtures), in which case the final state of the system is absent from equation (1) and the intermediate co-exists only with the initial state. An example of such an application is given by a study of nerve growth factor (NGF), a protein playing a key role in determining survival, differentiation and maintenance of specific neuronal populations during development. NGF is a functional homodimer composed of two non-covalently bound chains and its oligomerization state in solution is concentration dependent. A SAXS study (Covaceuszach *et al.*, 2015 ▸) demonstrated that NGF forms a mixture of dimers and head-to-head dimers of dimers. The system contained just two components and *DAMMIX *was applied to find an unknown shape of an NGF multimer given the known structure of an NGF dimer.

**Figure 4:**
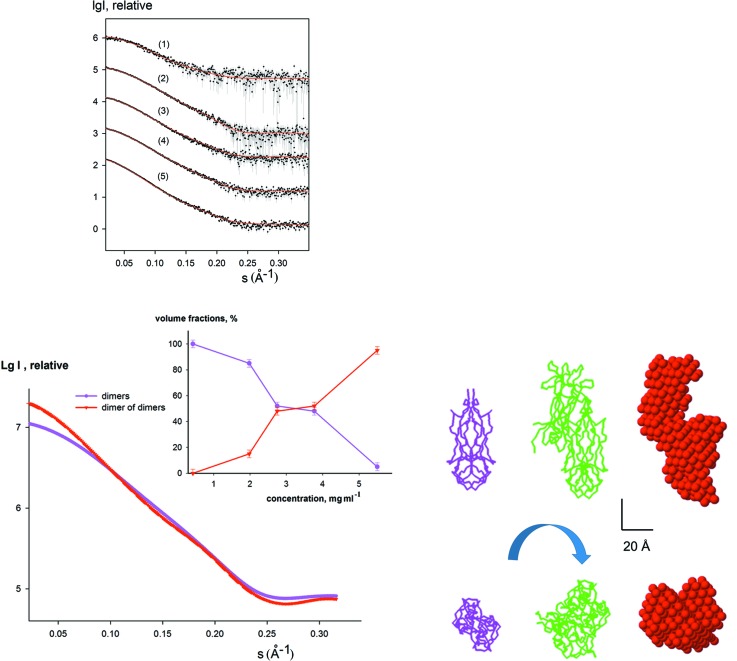
Analysis of NGF concentration-dependent oligomeric equilibrium [initial state; dimer model (PDB entry 1btg); intermediate state, unknown oligomer; final state, set to ‘none’]. The top left panel displays the experimental data (dots with error bars), and the *DAMMIX* fits as red solid lines. The previously reported models (dimer and head-to-head dimer of dimers) are shown in the bottom right panel with magenta and green C_α_ traces (Covaceuszach *et al.*, 2015 ▸). A typical restored shape of the unknown component obtained by *DAMMIX* is displayed in the bottom right panel with red beads. The scale bar is 20 Å. The scattering curves from the components are shown in the bottom left panel (the two most different restored curves for the intermediate obtained from multiple *DAMMIX* runs are shown with dashed red lines) and their restored volume fractions are displayed in the inset; the colours are the same as the bottom right panel and the error bars of volume fractions display the average dispersion over multiple *DAMMIX* runs.

The experimental curves from NGF solutions recorded in the concentration range from 0.43 to 5.5 mg ml^−1^ are displayed in Fig. 4 ▸. The scattering from the initial state was calculated from the dimeric PDB structure (PDB entry 1btg), and the ‘final’ state of the system (intensity and volume fractions of the third component, absent in this case) was set to zero. The reconstructed multimer was about twice the size compared to the ‘initial state’ dimer and had a shape similar to the head-to-head dimer-of-dimers structure reported by Covaceuszach *et al.* (2015 ▸). The restored volume fractions are also in good agreement with those reported in the previous SAXS study (Fig. 4 ▸, inset). The shape similarity of the restored dimer-of-dimers structure and average dispersion of volume fractions in the oligomeric mixture over multiple *DAMMIX* runs are shown in Table 1 ▸. These results also demonstrate that for two-component systems *DAMMIX* provides a meaningful solution and can be used for quantitative characterization of oligomeric equilibria, for example.

## 4. Discussion and conclusions   

Interpretation of the SAXS (and also of neutron scattering, SANS) data in terms of three-dimensional models is ambiguous for monodisperse systems (Petoukhov & Svergun, 2015 ▸) and even more so for mixtures. It is therefore not surprising that no methods have been available to directly restore the shape of an unknown component in an evolving system. Here, we consider a three-component system with one unknown intermediate and combine *ab initio* shape determination with a non-negative linear minimization for the component decomposition to reconstruct the shape of the intermediate and the volume fractions of the components. The method essentially works like a conventional shape determination with only two additional parameters (*i.e.* three volume fractions with the closure relationship *v*_m*k*_ + *v*_a*k*_ + *v*_i*k*_ = 1) while fitting multiple data sets. As demonstrated in several simulated and practical examples, the method does allow reliable shape reconstructions for different types of evolving systems by fitting the entire bulk of measured data.

It is clear that adequate tests (*e.g.* by running SVD) must be conducted prior to the utilization of *DAMMIX* to ensure that the system can indeed be represented by three major components. An example in the supporting information demonstrates what happens if one applies *DAMMIX* to a system with a larger number of evolving components. The process of α-synuclein fibrillation was shown to be described with four major components and the species contained, in addition to monomers and mature fibrils, also dimeric and oligomeric intermediates (Giehm *et al.*, 2011 ▸). As illustrated in Fig. S2 (see supporting information), *DAMMIX* returns a shape which lies in between those of dimeric and oligomeric intermediates, and misfits are also observed in some of the data sets indicating that the three-component description is not fully adequate. For some types of such multicomponent evolving systems it may be possible to select subsets of data where the SVD analysis detects three components, such that sequential analysis of the appropriate subsets would allow one to restore multiple unknown species. The chemometric (Herranz-Trillo *et al.*, 2017 ▸) and EFA approaches (Hopkins *et al.*, 2017 ▸) could be of great help in monitoring more sophisticated pathways of the kinetic processes and eventually finding subsets where *DAMMIX* can be applied.

The analysis of notoriously polydisperse systems is a highly complicated task and none of the available methods, each having their own limitations, provides a unique recipe to solve the ambiguity inherent in the SAXS data. For *DAMMIX*, which is an automated shape determination procedure, it should never be forgotten that an enantiomorphous structure yields the same scattering, and the enantiomorphs are also considered in all averaging and selection procedures. It is also important to remember that even a small contamination of the curve(s) of pure species can influence the shape reconstructions of the intermediates. To prevent such effects, potential impurities should be thoroughly checked by an estimation of the molecular weight and excluded volume of the sample as well as the linearity of the Guinier region (Guinier, 1939 ▸) and the shape of the *p*(*r*) function [in cases of contamination *p*(*r*) typically displays an additional tail at higher distances]. If high-resolution structures or models of pure species are available, they can be used as input for *DAMMIX *and the theoretical curves calculated by *CRYSOL* (Svergun *et al.*, 1995 ▸) can be employed as basic components of the mixture under study. Overall, the proposed method is complementary to the chemometric and EFA analysis, and the application of different methods is always recommended to increase confidence in the results.

The proposed method is also applicable for two-component evolving systems when one component (‘monomer’) is known, allowing one to restore the structure of the other one. This case can be considered a useful addition to the *GASBORMX* algorithm (Petoukhov *et al.*, 2012 ▸), which does not require the structure of the monomer to be known but is limited to symmetric protein oligomers, whereas the proposed approach is free from symmetry restrictions and can also be used for objects other than proteins.

*DAMMIX* is scheduled for inclusion in *ATSAS* release 2.9 (http://www.embl-hamburg.de/biosaxs/software.html), freely available to academic users. The running time of *DAMMIX* is similar to that of *DAMMIF* (within a few minutes on a standard workstation), and the program can be run from a command line making it applicable for automated data analysis pipelines. We expect that the new approach of model-free shape analysis of intermediates will be useful for the interpretation of a broad range of kinetic time-resolved SAXS/SANS experiments on evolving biological systems.

## Supplementary Material

Figure S1 and Figure S2. DOI: 10.1107/S2052252518005900/tj5014sup1.pdf

## Figures and Tables

**Table 1:** Results of multiple runs of *DAMMIX* (in all cases, 15 individual runs) for synthetic and experimental data sets The second column reports the average 〈NSD〉 values between pairwise superimposed models obtained from different *DAMMIX *runs for intermediate species; 〈Δ*v*_beg_〉, 〈Δ*v*_int_〉 and 〈Δ*v*_end_〉 are average dispersions of volume fractions for initial, intermediate and final states of the systems, respectively; 〈*N*_olig_〉 is the average oligomerization number for the intermediate species; 〈χ^2^〉 are the average differences of the restored curves from intermediate species obtained by *DAMMIX*, where the corresponding errors in brackets were estimated from the Poisson statistics.

Data set	〈NSD〉	〈Δ*v*_beg_〉, (%)	〈Δ*v*_int_〉, (%)	〈Δ*v*_end_〉, (%)	〈*N*_olig_〉	〈χ^2^〉	
Ellipsoid (Fig. 1 ▸)	0.92 (0.08)	1.9	2.1	1.6	5.1 (0.3)	1.13	
Insulin (Fig. 2 ▸)	0.87 (0.06)	1.8	2.2	1.5	4.6 (0.4)	1.05	
Lumazine synthase (Fig. 3 ▸)	0.94 (0.05)	1.79	2.4	1.8	N/A	1.03	
hNGF (Fig. 4 ▸)	0.85 (0.06)	1.6	1.7	N/A	2.1 (0.2)	1.04	
Cylinder (Fig. S1)	1.02 (0.09)	2.0	2.3	1.6	8.1 (0.2)	1.08	
α-Synuclein (Fig. S2)	1.22 (0.12)	2.2	2.5	1.9	8.3 (0.5)	1.37	
