# Supplementary material for: Direct shape determination of intermediates in evolving macromolecular solutions from small-angle scattering data
Source: IUCrJ. 2018 May 30;5(Pt 4):402–9. doi: 10.1107/S2052252518005900 (PMC6038953; doi:10.1107/S2052252518005900)
Supplement: Supplementary file 1 [file m-05-00402-sup1.pdf]

IUCrJ

Volume 5 (2018)

Supporting information for article:

Direct shape determination of intermediates in evolving macromolecular solutions from small-angle scattering data

Petr V. Konarev and Dmitri I. Svergun

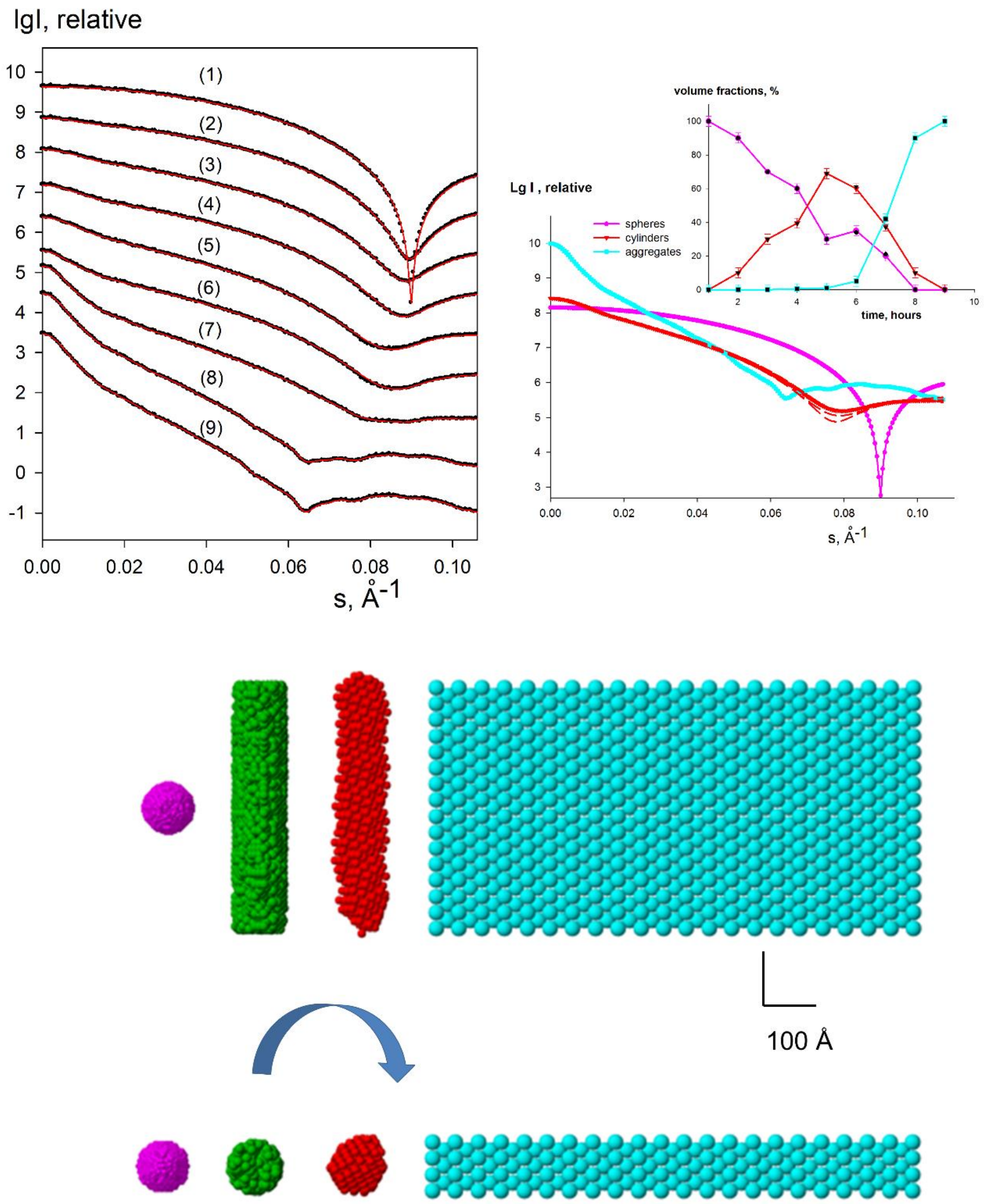

Figure S1 Simulated scattering curves from an evolving system (initial state, sphere with the diameter 100 Å, intermediate state, cylinder with the diameter 100 Å and height 500 Å, final state – rectangular prism with the side sizes 100, 500 and 1000 Å). The pure species curves from bead

models were calculated using DAMMIN, the linear combinations with the ‘designed’ volume fractions were generated using PRIMUS (Konarev et al., 2003). A relative error of 2% was added to the simulated data. In the top left panel, simulated data are shown as dots, the fits as red solid lines. The ideal shapes of the components (sphere, cylinder, rectangular prism) are shown in the bottom panel with magenta, green and cyan beads, respectively. A typical restored shape of the intermediate by DAMMIX is displayed in the bottom panel with red beads. The scale bar is 100 Å. The scattering curves from the components are shown in the top right panel (two most different restored curves for the intermediate obtained from multiple DAMMIX runs are shown with dashed red lines), their restored volume fractions are displayed in the insert (the colour notations are the same as for the bottom panel) together with the “designed” volume fractions (shown in black colour), the error bars of volume fractions display their average dispersion over multiple DAMMIX runs.

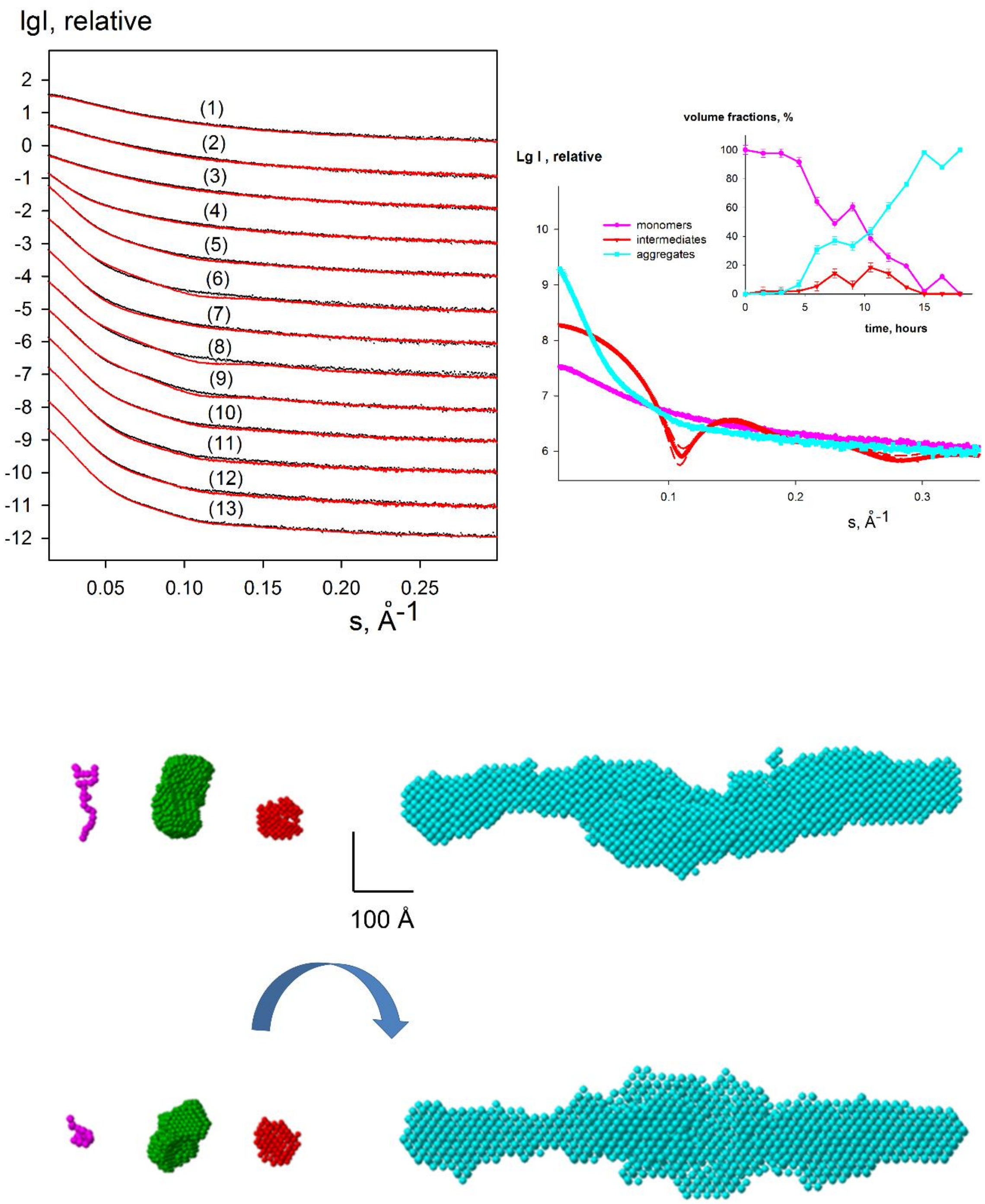

Figure S2 Analysis of intermediates during α-synuclein fibrillation. The initial state is represented by a monomer model of α-synuclein, and the final state by the bead model for the mature fibril (Giehm et al., 2011). Top left panel displays the experimental data (dots with error bars), and the DAMMIX fits as red solid lines. The shapes of the monomer and the aggregates are shown in the

bottom panel with magenta and cyan beads, respectively; the previously reported intermediate oligomer (16-mer) is displayed by green beads (Giehm et al., 2011). The presence of the forth component in the system is supported by singular value decomposition of SAXS data, and this component appears to contain dimeric species as was evidenced by gel filtration (Giehm et al., 2011). The scale bar is 100 Å. The restored shape of the intermediate obtained by DAMMIX displayed in the bottom panel (red beads) appears as about half that of the 16-mer being effectively an average of the two intermediates species (dimers and 16-mers) reported by (Giehm et al., 2011). The scattering curves from the components are shown in the top right panel (two most different restored curves for the intermediate obtained from multiple DAMMIX runs are shown with dashed red lines), their restored volume fractions are displayed in the insert, the colour notations are the same as for the bottom panel, the error bars of volume fractions display their average dispersion over multiple DAMMIX runs. Notably, DAMMIX fits reveal systematic deviations to experimental data at medium angles for several time points during fibrillation indicating that three components are not sufficient to adequately describe the system.
